# Comparison of next generation technologies and bioinformatics pipelines for capsular typing of *Streptococcus pneumoniae*

**DOI:** 10.1128/jcm.00741-23

**Published:** 2023-11-21

**Authors:** Desiree Henares, Stephanie W. Lo, Amaresh Perez-Argüello, Alba Redin, Pilar Ciruela, Juan Jose Garcia-Garcia, Pedro Brotons, Jose Yuste, Raquel Sá-Leão, Carmen Muñoz-Almagro

**Affiliations:** 1 Department of RDI Microbiology, Hospital Sant Joan de Déu, Barcelona, Spain; 2 Infectious Diseases and Microbiome, Institut de Recerca Sant Joan de Déu, Barcelona, Spain; 3 CIBER Center for Epidemiology and Public Health (CIBERESP), Instituto de Salud Carlos III, Madrid, Spain; 4 Parasites and Microbes Programme, Wellcome Sanger Institute, Hinxton, United Kingdom; 5 Milner Center for Evolution, Life Sciences Department, University of Bath, Bath, United Kingdom; 6 Surveillance and Public Health Emergency Response, Public Health Agency of Catalonia (ASPCAT), Barcelona, Spain; 7 Pediatrics Department, Hospital Sant Joan de Déu, Barcelona, Spain; 8 Department of Surgery and Medical-Surgical Specialties, Facultat de Medicina i Ciències de la Salut, Universitat de Barcelona, Barcelona, Spain; 9 School of Medicine, Universitat Internacional de Catalunya, Barcelona, Spain; 10 Spanish Pneumococcal Reference Laboratory, National Center for Microbiology, Instituto de Salud Carlos III, Madrid, Spain; 11 CIBER of Respiratory Diseases (CIBERES), Instituto de salud Carlos III, Madrid, Spain; 12 Laboratory of Molecular Microbiology of Human Pathogens, Instituto de Tecnologia Química e Biológica António Xavier, Universidade Nova de Lisboa (ITQB NOVA), Oeiras, Portugal; National Institute of Allergy and Infectious Diseases, Bethesda, Maryland, USA

**Keywords:** pneumococci, WGS, ONT, *in silico *serotyping, validation, Pathogenwatch

## Abstract

Whole genome sequencing (WGS)-based approaches for pneumococcal capsular typing have become an alternative to serological methods. *In silico* serotyping from WGS has not yet been applied to long-read sequences produced by third-generation technologies. The objective of the study was to determine the capsular types of pneumococci causing invasive disease in Catalonia (Spain) using serological typing and WGS and to compare the performance of different bioinformatics pipelines using short- and long-read data from WGS. All invasive pneumococcal pediatric isolates collected in Hospital Sant Joan de Déu (Barcelona) from 2013 to 2019 were included. Isolates were assigned a capsular type by serological testing based on anticapsular antisera and by different WGS-based pipelines: Illumina sequencing followed by serotyping with PneumoCaT, SeroBA, and Pathogenwatch vs MinION-ONT sequencing coupled with serotyping by Pathogenwatch from pneumococcal assembled genomes. A total of 119 out of 121 pneumococcal isolates were available for sequencing. Twenty-nine different serotypes were identified by serological typing, with 24F (*n* = 17; 14.3%), 14 (*n* = 10; 8.4%), and 15B/C (*n* = 8; 6.7%) being the most common serotypes. WGS-based pipelines showed initial concordance with serological typing (>91% of accuracy). The main discrepant results were found at the serotype level within a serogroup: 6A/B, 6C/D, 9A/V, 11A/D, and 18B/C. Only one discrepancy at the serogroup level was observed: serotype 29 by serological testing and serotype 35B/D by all WGS-based pipelines. Thus, bioinformatics WGS-based pipelines, including those using third-generation sequencing, are useful for pneumococcal capsular assignment. Possible discrepancies between serological typing and WGS-based approaches should be considered in pneumococcal capsular-type surveillance studies.

## INTRODUCTION

*Streptococcus pneumoniae* is a bacterial pathobiont that colonizes children’s and adults’ nasopharynx asymptomatically but can also cause localized infection and spread to sterile tissues causing invasive pneumococcal disease (IPD), mainly pneumonia, meningitis, bacteremia, and sepsis (1). It has been estimated that *S. pneumoniae* causes 300,000 annual deaths in children globally, despite the introduction of pneumococcal conjugate vaccines in the early 2000s (2).

Pneumococcal conjugate vaccines are designed against capsular polysaccharides of *S. pneumoniae* and produce a serotype-specific response in the host (3). This capsular polysaccharide, encoded by the capsule polysaccharide locus (*cps* locus), is the main virulence factor of this bacterium (4). More than 100 serotypes have been identified to date according to antigenic properties of the capsule (5), although only the most prevalent serotypes causing IPD have been included in the formulations of these vaccines (6).

Multi-valent pneumococcal conjugate vaccines successfully achieved their objective, dramatically reducing the morbidity and mortality of IPD in comparison to the pre-vaccine era (2, 7). However, continuous surveillance of pneumococcal serotypes causing IPD in multiple countries has demonstrated a phenomenon of serotype replacement due to the increase of IPD by non-vaccine serotypes. These pneumococcal strains can be virulent and multi-drug resistant (7 – 9). Of note, differences in serotype distribution have also been described according to geographical area (10, 11). Thus, serotyping of pneumococcus causing IPD is essential to monitor vaccine effectiveness, emergence and dissemination of antimicrobial resistance, investigate new vaccine strategies, and address specificities of vaccine programs according to local epidemiology.

The current gold standard technique for pneumococcal serotyping is the Quellung reaction, based on a reaction of capsular polysaccharide with homologous anticapsular antibody, and its examination under the microscope for observation of bacterial swelling (12, 13). Other phenotypic methods based on anticapsular antisera including dot blot and latex agglutination assays have also emerged to complement this technique (14, 15). However, these phenotypic methods may be labor intensive and time-consuming, especially for large studies, require a significant amount of sera, and/or are prone to subjectivity in unexperienced hands (16, 17).

Next-generation sequencing (NGS) combined with different bioinformatics approaches is revolutionizing molecular epidemiology surveillance. The appearance of high throughput sequencing and the possibility to perform whole genome sequencing (WGS) has contributed to an increasing number of *in silico* approaches to detect the *cps* locus of *S. pneumoniae* and predict the corresponding serotype from WGS data (18 – 20). Moreover, WGS allows the detection of all described serotypes by common *in silico* serotyping approaches and the identification of potentially new ones, or even genetic variants of known serotypes, which could or could not be relevant to phenotype when further analyses are performed on the *cps* sequence (21).

At present, NGS technologies that generate short reads are considered as a common tool for microbial genomics due to their high throughput with high accuracy. They have, however, the drawback of requiring large bench space, the relatively high cost of the sequencing platforms, and the need, for most laboratories, of processing a large number of samples in a single batch to be cost effective (22). In contrast, some long-read sequencing platforms, such as the MinION sequencer from Oxford Nanopore Technologies (ONT), constitute a revolutionary technology; a scalable, portable, and low-price platform that enables the sequencing of a reduced number of samples per batch allowing for major flexibility in terms of delivering results for surveillance and epidemiological purposes (23, 24). Although with lower precision than Illumina, this technology could be very useful for determining the complete sequences and organization of genes in operons of interest such as the *cps* locus from *S. pneumoniae*.

Most common and already validated bioinformatics programs for *in silico* pneumococcal serotyping from WGS data derived from pure bacterial cultures include PneumoCaT, SeroBA and the pipeline developed at the U.S. CDC (Centers for Disease Control and Prevention) (18 – 20), which are mainly designed for directly working with fastq reads from short-read sequencing approaches. The PneumoCaT pipeline uses a two-step approach. First, it maps fastq paired-end reads to a database of capsular locus sequences for all known capsular types and predicts a serotype if a single *cps* locus with >90% of coverage is matched, and this *cps* locus does not belong to a genogroup. Second, if ambiguous identification occurs or the serotype belongs to a genogroup, a second variant-based step is performed using a database that contains previously described capsular genetic variants that can discriminate serotypes within a serogroup/genogroup (19). SeroBA follows a similar approach but works with *k*-mers, which makes it computationally more efficient (18). Both constitute command-line programs that require management with a linux environment and some bioinformatics skills for launching these programs.

In this regard, Pathogenwatch is a platform for genomic surveillance of microbial pathogens that can assign pneumococcal serotypes using an online web tool with a user-friendly interface (25), thus being especially useful for laboratories with less bioinformatics expertise. SeroBA is built in within the pneumococcal genome analysis workflow on Pathogenwatch. Pathogenwatch can rapidly predict pneumococcal serotypes directly from either assembled genomes or fastq reads, as opposite to SeroBA command-line version which requires fastq reads as input (26). When assembly is submitted for serotype prediction on Pathogenwatch, pIRS (profile-based Illumina pair-end reads simulator) is used to simulate pair-end reads as input for SeroBA. Allowing assembled genomes as an input is another attractive characteristic of Pathogenwatch that paves the way to *in silico* predict pneumococcal serotypes from other type of sequencing data, as those produced by long-read sequencing technologies.

In this report, we aimed to determine the capsular types of pneumococci causing IPD at Hospital Sant Joan de Déu (HSJD) between 2013 and 2019 using both serological typing and *in silico* serotyping with WGS. In addition, we aimed to compare the performance of the *in silico* programs described using two different approaches: short-read sequencing of pneumococcal isolates followed by serotyping with PneumoCaT, SeroBA, and Pathogenwatch vs long-read sequencing by MinION-ONT coupled with serotyping by Pathogenwatch from pneumococcal assembled genomes.

## MATERIALS AND METHODS

### Study design

This study used a large collection of pneumococcal isolates stored in the laboratory biobank of the Research and Innovation Microbiology Department located at University Hospital Sant Joan de Déu (HSJD) in Esplugues, Barcelona (Spain). This department was designated in the year 2009 as a reference center for molecular epidemiological surveillance of IPD in Catalonia by the Public Health Agency of Catalonia. All health centers from Catalonia are invited (not compulsory) to send their pneumococcal isolates for molecular characterization. For this study, we selected all pneumococcal isolates obtained from normally sterile samples (blood, pleural fluid, cerebrospinal fluid, and synovial fluid) of children <18 years who attended HSJD from January 2013 to December 2019. Pneumococcal isolates were stored in preservation media of skimmed milk and stored at −80°C.

### Serological typing based on the use of anticapsular antisera

**Fig 1:**
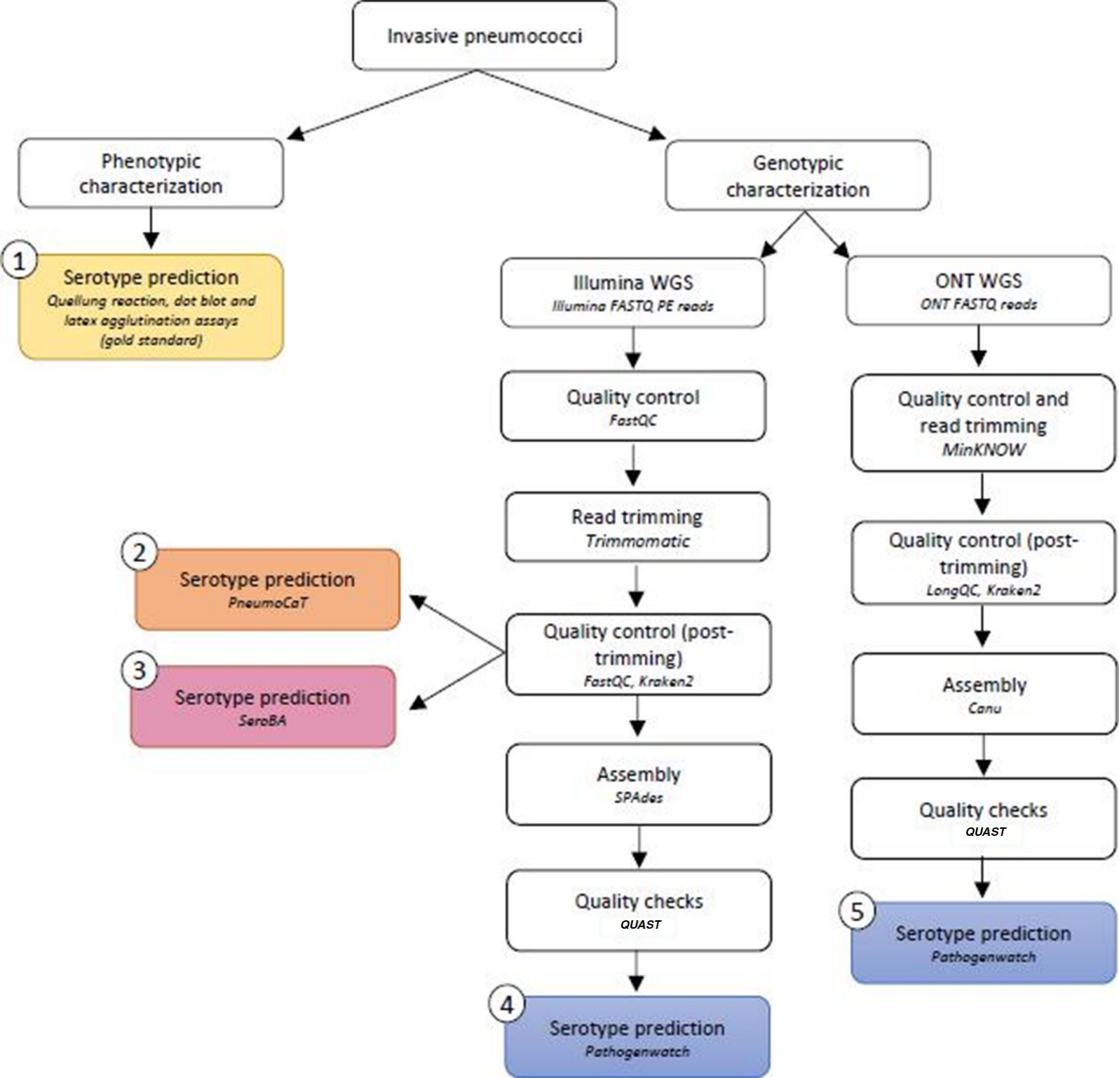
Workflow for pneumococcal serotyping pipelines.

All clinical isolates were serologically typed using dot blot (14), Quellung reaction with pneumococcal factor antisera (Statens Serum Institut, Copenhagen, Denmark) and Immulex Pneumotest kit (Statens Serum Institut, Copenhagen, Denmark) at the National Center for Microbiology (Majadahonda, Madrid, Spain; Fig. 1). These phenotypic methods were considered the gold standard for comparisons performed in this manuscript.

### Pneumococcal serotyping using WGS

Different genotypic approaches were considered using WGS on pneumococcal isolates coupled with *in silico* serotyping. Two different sequencing methodologies were utilized; a short-read approach performed by Illumina platforms and a long-read approach carried out with ONT technology (Fig. 1).

#### Short-reads by Illumina sequencing

Pure cultures from each pneumococcal isolate (kept in skimmed milk at 80°C) were grown overnight on blood agar plates at 37°C with a 5% CO_2_-enriched atmosphere in the HSJD laboratory. Cells from pure cultures were resuspended in DNA/RNA shield (Zymo Research, USA) at a 0.5 McFarland, which corresponds to around 10^8^ colony forming units/mL, and were sent to MicrobesNG or Sanger Institute facilities to perform DNA extraction, library preparation, and sequencing as previously described (https://www.microbesng.com) (8). Bioinformatics analyses including *in silico* serotyping were carried out at HSJD using the raw fastq files provided.

##### Bioinformatics analyses and *in silico* serotyping

The quality of raw reads was initially assessed with FastQC v0.11.9 (27). Reads were trimmed with Trimmomatic v0.39 (28) using an average sliding window quality cut-off of Q15 and discarded if they presented a minimum length lower than 15% of the total read length amplicon after trimming. Quality control with FastQC was additionally performed at this point. Taxonomic assignment of Illumina reads was carried out with Kraken2 v2.0.8-beta (29) in order to evaluate the presence of contaminant sequences. Quality-trimmed paired-end reads were the input for serotyping using PneumoCaT v1.2.1 (Illumina-PneumoCaT pipeline) (19) and SeroBA v1.0.2 (Illumina-SeroBA pipeline) (18). *De novo* assembly was performed on quality-trimmed reads using SPAdes v3.13.1 (30) prior to *in silico* serotyping with Pathogenwatch (Illumina-Pathogenwatch pipeline) (25). Basic statistics for assembled genomes were computed with QUAST (31) (Fig. 1). Primary metrics of Illumina sequencing data and assembled genomes have been included in Supplementary material 1.

### Long-reads by pocket-size MinION-ONT sequencing

DNA extraction with ZymoBIOMICS DNA Microprep kit (ZYMO RESEARCH), library preparation with the Rapid Barcoding Kit (SQK-RBK004) (ONT), and WGS with the MinION device (ONT) on an R9 flow cell (FLO-MIN106; ONT) was performed at HSJD as previously described, with a simple and rapid workflow of about 21 hours for up to 12 genomic DNA samples (23).

#### Bioinformatics analyses and *in silico* serotyping

MinKNOW (version 21.06.2/21.10.4) is the operating software of ONT devices that performed data acquisition, quality control, and real-time FAST basecalling, which ensures high performance of 92% of accuracy. Briefly, raw data signals from the sequencer were saved in FAST5 files. Real-time basecalling with Guppy (integrated into MinKNOW) converted the FAST5 files to fastq. Demultiplexing and quality control were also performed with MinKNOW by discarding reads with mean quality lower than 7. Quality control of ONT reads was additionally performed with longQC (32). Taxonomic assignment of ONT reads was carried out with Kraken2 v2.0.8-beta in order to evaluate the presence of contaminants. Finally, ONT reads were *de novo* assembled by Canu v1.9 (33) with default parameters prior to pneumococcal *in silico* serotyping using the online web-tool Pathogenwatch based on the SeroBA program (v1.0.1; ONT-Pathogenwatch pipeline) (25). Basic statistics of ONT assembled genomes were calculated by QUAST (Fig. 1). Primary metrics of ONT sequencing data and assembled genomes have been included in Supplementary material 1.

### Concordance between pneumococcal serotyping approaches

Percentages of concordance were calculated for genotypic approaches in comparison to serological typing. Some considerations were followed for concordance analyses; first, no genetic differences have been consistently detected for distinguishing serotypes within serogroup 24, so genotypic approaches can only predict up to serogroup level (19, 34). Second, 15B and 15C serotypes, as well as 35B and 35D serotypes, are difficult to distinguish using common serological methods in the laboratory and can present interconversion events (35 – 38). Therefore, they were grouped as 15B/C and 35B/D for the purpose of the study as previously done (36). In addition, Adjusted Rand measured the overall agreement of two-typing methods considering that agreement of partitions could arise from chance and applying a correction.

### Detailed analyses of discrepant results

PROKKA v1.14.16 (39) was used to annotate Illumina and ONT assemblies, and ARTEMIS (40) was used to extract capsular locus sequences from these assemblies by locating *aliA* or *dexB* flanking regions (41). Query *cps* sequences and reference *cps* sequences were aligned using Geneious Prime 2023.0.1 and the Mauve algorithm (https://www.geneious.com). Reference sequences for capsular types were retrieved from NCBI (http://www.ncbi.nlm.nih.gov/) and were those published by reference (41) and utilized by SeroBA (18). Query coverage and percent of the identity of the capsular locus with the reference sequences of discrepant serotypes were extracted. If matching to a single of the discrepant reference capsular sequences with coverage >90%, the capsular type was confirmed. If matching to two reference sequences of discrepant serotypes with coverage >90%, a variant-based approach was performed, similar to that performed in PneumoCaT and SeroBA pipelines (Supplementary material 2). Briefly, previously aligned serotype-specific genes were further inspected for detecting specific variants that are commonly used to differentiate between genetically related serotypes, including SNPs (single nucleotide polymorphisms) and deletions. These distinctive genetic features are the same as described in SeroBA and PneumoCaT variant analyses (Supplementary material 3A). Additional variants were inspected in order to detect other changes that could be contributing to serotyping mispredictions on these samples. A gene was defined as present in the assembly if a minimum sequence identity of 90% and alignment coverage of 95% was determined. Finally, Illumina and ONT fastq reads were mapped to the reference serotype specific-gene sequences in order to extract coverage and variant frequency.

## RESULTS

A total of 128 pneumococcal isolates were obtained from 128 invasive samples (blood, *n* = 92; cerebrospinal fluid, *n* = 16; pleural fluid, *n* = 13; and synovial fluid, *n* = 7) during the study period. These 128 isolates caused 121 episodes of IPD: 47, 42, 20, 8, and 4 episodes of pneumonia, bacteraemia without focus, meningitis, septic arthritis, and sepsis, respectively, in 119 patients (two patients had two episodes of IPD more than 3 months apart). The median patient’s age at the episode development was 2 years (interquartile range: 0.04–4.02), and about half of the episodes occurred in the male population (52.9%). From these 128 isolates, 121 were selected for sequencing (one per episode), and 119 were available for WGS (two isolates were lost due to poor preservation through time) and were finally included in the present study.

Among the 119 isolates, a total of 29 different serotypes were detected by serological typing methods, and the most frequent were 24F (*n* = 17), 14 (*n* = 10), 15B/C (*n* = 8), 1 (*n* = 7), 3 (*n* = 7), and 8 (*n* = 7; Table 1). Overall a high percentage of concordance between serologically derived serotypes and *in silico* predicted serotypes from WGS data was observed, with over 91% of the isolates being assigned with the same serotype regardless of the method used (Table 1). Additionally, Adjusted-Rand also identified a high degree of agreement between genotypic and phenotypic methods and demonstrated a similar performance for all of them (Supplementary material 3B). The first analysis showed that Illumina-SeroBA and Illumina-Pathogenwatch pipelines were the methods with the highest concordance (98.3% for both, *n* = 117/119) when compared to the serological serotypes. Illumina sequencing followed by *in silico* serotyping with PneumoCaT exhibited a 94.1% (*n* = 112/119) concordance with serological serotypes. This slightly lower value was due to the presence of five discrepant results, as well as to the inability to predict a serotype in two samples that did not meet the requirements of quality metrics established by PneumoCaT. Finally, ONT sequencing coupled with Pathogenwatch pipeline had 91.6% (*n* = 109/119) agreement with serological types (Table 1).

**TABLE 1:** Comparison of genotypic approaches for pneumococcal serotyping from WGS data

	Pipeline	PneumoCaT (command-line program)		SeroBA (command-line program)		Pathogenwatch (graphic user interface web application)			
	Input data	Illumina fastq reads		Illumina fastq reads		Illumina-assemblies		ONT-assemblies	
Serological serotype	*N* ^*c*^	Concordant	Discordant/failed	Concordant	Discordant	Concordant	Discordant	Concordant	Discordant
Serogroup 24^*a*^	17	17		17		17		17	
14	10	10		10		10		10	
15B/C	8	8		8		8		8	
1	7	5	2^*b*^	7		7		7	
3	7	7		7		7		7	
8	7	7		7		7		7	
10A	6	6		6		6		6	
15A	6	6		6		6		6	
19A	5	5		5		5		5	
19F	5	5		5		5		5	
12F	4	4		4		4		4	
22F	4	4		4		4		4	
33F	4	4		4		4		4	
38	4	4		4		4		4	
18C	3		3	2	1	2	1		3
23B	3	3		3		3		3	
35B/D	2	2		2		2		2	
9V	2	2		2		2			2
11A	2	2		2		2			2
16F	2	2		2		2		2	
23A	2	2		2		2		2	
27	2	2		2		2		2	
4	1	1		1		1		1	
6A	1	1		1		1			1
6B	1	1		1		1		1	
6C	1		1	1		1			1
13	1	1		1		1		1	
29	1		1		1		1		1
35F	1	1		1		1		1	
Total	119	112	7	117	2	117	2	109	10
Concordance (%)		94.1		98.3		98.3		91.6	

^*a*^ Genotypic approaches predicted up to serogroup level for all isolates identified as serotype 24F by Quellung reaction (*n* = 17).

^*b*^ PneumoCaT applies a quality metric, which requires a mean depth of 20 reads across mapped sequence and a minimum depth of 5 reads for mapping. If these conditions are not met, PneumoCaT fails to predict the serotype. In this case, PneumoCaT failed to predict a serotype for two isolates that were typed as serotype 1 by serological methods.

^*c*^ *N*; sample size.

The main initial discrepancies between phenotypic and genotypic approaches were at the serotype level within a given serogroup (Supplementary material 3C). All genotypic approaches showed discrepancies for serogroup 18, misidentifying one 18C serotype as 18B. In the case of PneumoCaT pipeline, two other isolates typed as 18C by phenotypic methods were mispredicted as 18B serotypes by this pipeline, as well as one 6C serotype as 6D. Regarding ONT-Pathogenwatch pipeline, this method showed all previously described discrepancies as well as additional mispredictions within serogroups 6, 9, and 11 (Supplementary material 3C). Briefly, serological types 6A, 6C, 9V, and 11A were predicted as serotypes 6B, 6D, 9A, and 11D, respectively, by ONT-Pathogenwatch method. To be noted, and interestingly, only one misprediction was observed at the serogroup level; all *in silico* serotyping approaches assigned a serotype 35B/D to a serotype 29 determined by serological testing (sample 3855).

Among the 10 samples with discrepant results, further detailed analysis of discrepancies was performed by investigating pneumococcal assembled genomes using Illumina and ONT WGS data. Table 2 summarizes the results for analyses performed on Illumina and ONT-assembled genomes, and the final serotypes predicted according to the variants detected in these key genes. A manual inspection of serotype-specific genes allowed detection of distinctive genetic features between serotypes, as previously described (Supplementary material 3A). Moreover, additional presumed variants not previously described were detected among these serotype-specific genes, especially among ONT *cps* assembled sequences. These newly presumed variants could be contributing to mispredictions when using ONT-Pathogenwatch pipeline for pneumococcal typing of serotypes from serogroups 6, 9, 11, and 18. These presumed variants were essentially deletions on homopolymeric regions of these key *cps* genes which led to frame shifts, resulting in early stop codons and truncated genes on ONT *cps* sequences (Supplementary material 4). Interestingly, such presumed variants were not detected on the corresponding Illumina *cps* assembled sequences (Supplementary material 4) or directly from Illumina fastq reads (Supplementary material 3D). To be noted, only one new presumed variant (sample 1963) was found in an Illumina *cps* sequence which determined a 18B serotype in contraposition to the prediction of 18C serotype by serological tests. The presence of this new presumed variant was also confirmed on the ONT *cps* sequence (Supplementary material 4) and fastq reads obtained from this sample (Supplementary material 3D). Therefore, among the 10 samples with discrepant results (Table 2), 8/10 discrepancies were resolved after manual inspection of *cps* sequences derived from Illumina and none from ONT. Overall, the *cps* locus extracted from Illumina-assembled genomes exhibited a higher percent of identity to the reference capsular locus and specific-serotype genes than *cps* sequences extracted from ONT assemblies. Finally, the isolate that showed a misprediction at the serogroup level was re-typed by serological testing obtaining the same assignment to serotype 29 (sample 3855). Subsequently, for comparison of genetic identity, the *cps* locus sequence was extracted from the Illumina and ONT-assembled genomes and aligned to the reference *cps* sequences for serotypes 35B/D and serotype 29. A higher identity of the *cps* sequence to the reference sequences of 35B/D serotypes was confirmed, observing alignment coverages of 100% with identities over 99%. In contraposition, an alignment coverage of 74% and a percent of identity lower than 84% was observed for the alignment to the reference sequence of serotype 29 (Table 2).

**TABLE 2:** Genetic variants detected on Illumina and ONT-assembled genomes^*a*^

Sample	Serological serotype	Discordant predicted serotype (pipeline)	Assembled-genome	Reference sequences	Alignment identity (%)	Alignment query cover (%)	Variants detected	Functional effect	Final serotype predicted
2816	6A	6B (ONT-Pathogenwatch)	Nanopore	6A-CR931638	98.37	100.00	277–278delTT*	Frame shift leading to early stop codon (95)	6B
				6B-CR931639	98.36	93.00			
				*wciP* (6A)	99.39	100.00			
				*wciP* (6B)	99.19	100.00			
			Illumina	6A-CR931638	99.08	100.00	Intact *wciP*		6A
				6B-CR931639	99.07	93.00			
				*wciP* (6A)	99.90	100.00			
				*wciP* (6B)	99.70	100.00			
3012	6C	6D (Illumina-PneumoCaT, ONT-Pathogenwatch)	Nanopore	6C-EF538714	98.95	99.00	225delT*	Frame shift leading to early stop codon (79)	6D
				6D-GQ848645	98.38	100.00			
				*wciP* (6C)	99.49	100.00			
				*wciP* (6D)	98.58	100.00			
			Illumina	6C-EF538714	99.43	99.00	584 A > G	Aminoacid substitution (Ser195Asn) which results to different rhamnose-ribitol (1→3)	6C
				6D-GQ848645	98.86	100.00			
				*wciP* (6C)	100.00	100.00			
				*wciP* (6D)	99.09	100.00			
3052	9V	9A (ONT-Pathogenwatch)	Nanopore	9A-CR931645	98.98	100.00	107delT*	Frame shift leading to early stop codon (47)	9A
				9V-CR931648	98.97	100.00			
				*wcjE* (9A)	98.07	100.00			
				*wcjE* (9V)	97.98	100.00			
			Illumina	9A-CR931645	99.65	100.00	Intact *wcjE*		9V
				9V-CR931648	99.64	100.00			
				*wcjE* (9A)	99.13	100.00			
				*wcjE* (9V)	99.23	100.00			
3077	9V	9A (ONT-Pathogenwatch)	Nanopore	9A-CR931645	98.93	100.00	107delT*	Frame shift leading to early stop codon (47)	9A
				9V-CR931648	98.92	100.00			
				*wcjE* (9A)	98.26	100.00			
				*wcjE* (9V)	98.16	100.00			
			Illumina	9A-CR931645	99.60	100.00	Intact *wcjE*		9V
				9V-CR931648	99.59	100.00			
				*wcjE* (9A)	99.52	100.00			
				*wcjE* (9V)	99.61	100.00			
2508	11A	11D (ONT-Pathogenwatch)	Nanopore	11A-CR931653	99.15	100.00	10delA*	Frame shift leading to early stop codon (20)	11D
				11D-CR931656	99.30	100.00			
				*wcrL* (11A)	99.31	100.00			
				*wcrL* (11D)	99.31	100.00			
			Illumina	11A-CR931653	99.80	100.00	Intact *wcrL*		11A
				11D-CR931656	99.97	100.00			
				*wcrL* (11A)	99.86	100.00			
				*wcrL* (11D)	99.86	100.00			
2112	11A	11D (ONT-Pathogenwatch)	Nanopore	11A-CR931653	99.10	100.00	10delA*	Frame shift leading to early stop codon (20)	11D
				11D-CR931656	99.25	100.00			
				*wcrL* (11A)	99.31	100.00			
				*wcrL* (11D)	99.31	100.00			
			Illumina	11A-CR931653	99.74	100.00	Intact *wcrL*		11A
				11D-CR931656	99.92	100.00			
				*wcrL* (11A)	99.86	100.00			
				*wcrL* (11D)	99.86	100.00			
1963	18C	18B (Illumina-PneumoCaT, Illumina-SeroBA, Illumina-Pathogenwatch, ONT-pathogenwatch)	Nanopore	18B-CR931672	99.17	100.00	22delA* 436delT*	Frame shift leading to early stop codon (18)	18B
				18C-CR931673	99.17	100.00			
				*wciX* (18B)	98.70	100.00			
				*wciX* (18C)	98.80	100.00			
			Illumina	18B-CR931672	99.98	100.00	436delT*	Frame shift leading to early stop codon (158)	18B
				18C-CR931673	99.98	100.00			
				*wciX* (18B)	99.80	100.000			
				*wciX* (18C)	99.90	100.00			
3742	18C	18B (Illumina-PneumoCaT, ONT-Pathogenwatch)	Nanopore	18B-CR931672	99.31	100.00	96delT*	Frame shift leading to early stop codon (43)	18B
				18C-CR931673	99.31	100.00			
				*wciX* (18B)	98.20	100.00			
				*wciX* (18C)	98.30	100.00			
			Illumina	18B-CR931672	99.98	100.00	Intact *wciX*		18C
				18C-CR931673	99.98	100.00			
				*wciX* (18B)	99.90	100.00			
				*wciX* (18C)	100.00	100.00			
2291	18C	18B (Illumina-PneumoCaT, ONT-Pathogenwatch)	Nanopore	18B-CR931672	99.36	100.00	22delA*	Frame shift leading to early stop codon (18)	18B
				18C-CR931673	99.36	100.00			
				*wciX* (18B)	98.90	100.00			
				*wciX* (18C)	99.00	100.00			
			Illumina	18B-CR931672	99.98	100.00	Intact *wciX*		18C
				18C-CR931673	99.98	100.00			
				*wciX* (18B)	99.90	100.00			
				*wciX* (18C)	100.00	100.00			
3855	29	35B/D (Illumina-PneumoCaT, Illumina-SeroBA, Illumina-Pathogenwatch, ONT-pathogenwatch)	Nanopore	35B-CR931705	99.29	100.00			35B/D
				35D-KY084476	99.27	100.00			
				29-CR931694	83.31	74.00			
			Illumina	35B-CR931705	99.88	100.00			35B/D
				35D-KY084476	99.87	100.00			
				29-CR931694	83.64	75.00			

^*a*^ *Presumed variants detected on Illumina and/or ONT-assembled genomes

## DISCUSSION

In this study, we have determined the capsular types of pneumococcal isolates causing IPD in HSJD between 2013 and 2019. IPD occurred in children with a median age of 2 years, and about 50% of the episodes occurred in the male population. Serotype 24F was the main capsular type detected (14.3%), followed by 14 (8.4%) and 15B/C (6.7%), which is in line with the epidemiology of pneumococcus reported in the region of Catalonia in children under 5 years of age (9).

These serotypes were determined using both serological typing assays as well as different bioinformatics tools for assigning pneumococcal serotypes directly from WGS data obtained from Illumina and ONT sequencers. The most common pipelines utilized for pneumococcal serotyping from Illumina WGS data (PneumoCaT, SeroBA, and Pathogenwatch) were included in the analyses, as well as a new approach based on ONT WGS data and Pathogenwatch. The comparison of these methods demonstrated the usefulness of WGS-based approaches for capsular typing independently of the sequencing platform and bioinformatics pipeline utilized, as showed by an initial concordance with serological results of over 91% accuracy for all genotypic approaches. The Illumina-SeroBA and Illumina-Pathogenwatch were the pipelines with the best results, followed by Illumina-PneumoCaT and ONT-Pathogenwatch. Overall, these results are in line with those of the original publications that validated PneumoCaT, SeroBA, and Pathogenwatch using Illumina WGS data (18, 19, 25), with similar levels of concordance with the phenotypic assays. However, to our knowledge, there are no reports that have validated the use of the Pathogenwatch pipeline to nanopore sequencing data for pneumococcal serotype prediction. A single previous pilot study from our group suggested the usefulness of ONT for this purpose in a reduced number of samples (23). The current study expands the number of samples, validates this approach, and identifies highly genetically related serotypes among which the pipeline may fail to make correct predictions probably due to sequencing errors in homopolymeric regions.

Despite the overall high concordance of all WGS-based approaches with serological types, some discrepancies were noted. The ONT-Pathogenwatch was the pipeline with a higher number of discrepant results in comparison to serological testing (*n* = 10). These discrepancies were mainly observed within the serogroup level: 6A/B, 6C/D, 9A/V, 11A/D, and 18B/C. Distinctive genetic features between these serotypes mainly rely on single SNPs and single nucleotide deletions at just one gene resulting in differential functional effects (42 – 46). Altogether, these results suggest that special care must be taken if considering genetically closely related serotypes when using ONT-Pathogenwatch, but assignments can be made with confidence between highly distinctive *cps* sequences.

Interestingly, the discrepancies observed between ONT-Pathogenwatch and serological reactions in genetically related serotypes were due to the presence of deletions in homopolymeric regions which altered the reading frame and coded for early stop codons, modifying the functionality of these genes. It has been described that about half of the errors detected in ONT sequencing data are due to the presence of homopolymers. Generally, homopolymeric regions tend to be underestimated resulting in many deletion errors, as a consequence of the ONT sequencing chemistry and its reading software. Changes in the electric signal are detected when nucleotidic bases pass through the channel; therefore, the basecalling software is more prone to error when the same nucleotidic bases are repeated several times (47). Our findings also support the hypothesis that nanopore sequencing errors may be involved in our serotype mispredictions with the ONT-Pathogenwatch pipeline. In this regard, deletions found in ONT-assembled genomes were not detected in the corresponding Illumina assemblies, and correct predictions using Illumina data were obtained through SeroBA and Illumina-Pathogenwatch pipelines. Therefore, original discrepancies for ONT-Pathogenwatch may be more driven by the sequencing technology itself than by the real presence of new variants.

Only one discrepancy observed in an 18C serotype predicted by the serological reaction was mispredicted as 18B by all genotypic approaches. A 436delT in the *wciX* gene was found in the *cps* sequence of this isolate. This deletion altered the reading frame and coded for an early stop codon which truncated the gene, as occurs in 18B serotypes. This variant was found in *cps* sequences derived from both Illumina and ONT assemblies and confirmed its presence in raw reads with high coverage and frequency. These findings may support a serological cross-reaction between 18B/C serotypes as a possible origin for this discrepancy, instead of WGS or WGS-pipeline assignment errors. However, we did not repeat serological testing for this sample. In fact, a limitation of this work may include the not repeatability of the serological testing for most discrepant samples, which helped to resolve some discrepant results in a previous publication (19).

Another important discrepant result was reported between serotype 29 and serotype 35B/D. Serological typing determined a serotype 29 in one pneumococcal isolate, while all WGS-based approaches determined a 35B/D *cps* locus. Large differences in their genetic sequences have been detected between both serotypes, so both sequencing technologies have enough capacity to reproduce these differences and discriminate between these serogroups, pointing to another origin rather than genetic for the discrepancy observed. In this regard, serotype 35D shows serological reactivity and chemical structure similar to that of serotype 29. In fact, cross-reactivity of serotype 29 with factor sera for group 35 has been reported (19, 35). Thus, both serotypes are difficult to distinguish by serological methods. Analyses including more clinical isolates typed as serotype 29 by serological methods vs WGS would be of interest in future-related studies.

At present, there is an increasing number of reports that have utilized WGS for surveillance of capsular types of *S. pneumoniae* causing IPD (21, 48 – 51), but this methodology has not been widely adopted yet (52 – 56). These may be in part due to the lack of accessibility of Illumina sequencing to medium and small-size laboratories, making it necessary to send the samples to reference laboratories, as well as to the fact that most programs utilized for *in silico* serotyping require some bioinformatics skills not available in all laboratories (18, 19). In this regard, the validation of the online and user-friendly pipeline of Pathogenwatch coupled with ONT sequencing is an important milestone. This rapid and simple workflow can manage 12 samples in just 24 hours and can greatly decrease time-to-results and acts as a bridge to clinical and epidemiological settings. We have shown that high levels of accuracy, as high as 91.6%, are associated to this automated method.

The WGS-based approaches are very useful for pneumococcal characterization. The pneumococcus is known for its high genetic diversity and the ability to do capsular switching (41, 57, 58). Characterizing the *cps* sequence is essential for the surveillance of pneumococcal capsular types and informing vaccine policy-makers. Moreover, WGS approaches can give us information not only from the capsule type but also they can give us much more information on other aspects related to their virulence, resistance patterns, and other key aspects of pneumococcus (8, 59). However, we must consider that WGS-based approaches may not replace the serological typing reaction. Although serological typing methods are time-consuming when testing large number of samples, require expertise, and are prone to subjectivity in unexperienced hands, phenotypic approaches are still valid. In this regard, some serotypes included in the current vaccines can only be resolved according to serological testing due to the lack of distinctive genetic features at the time of writing this report, as serotypes from serogroup 24 (19). Although some genetic differences have been recently detected that could help to predict these serotypes in the future (34), these differences were not consistently detected among all strains. Moreover, a relatively small number of strains were tested, which may not form a representative sample for validation of these genetic variants. In addition, nanopore long-read sequencing for genotypic determination of serotypes can be challenging when trying to differentiate among highly genetically related serotypes due to the lower accuracy of this technology; therefore, confirmation by phenotypic testing may be necessary. Finally, it is important to note that a serotype is primarily based on the serological immune response, and the genetic basis can be used as a proxy for the serotype. Therefore, these suggest that a combination of serological typing with WGS-based approaches can be especially useful for surveillance laboratories.

In conclusion, our study demonstrates that WGS-based approaches coupled with different bioinformatics pipelines are useful tools for pneumococcal capsular assignment, including those using third-generation sequencing. Possible discrepancies between serological testing and WGS-based approaches should be taken into account in pneumococcal capsular typing.

## Data Availability

The authors confirm all supporting data and protocols have been provided within the article or through supplementary data files. Raw sequence files have been deposited in the European Nucleotide Archive (ENA) at EMBL-EBI under BioProject accession number PRJEB57405.

## SUPPLEMENTAL MATERIAL

Supplementary material 1 jcm.00741-23-s0001.xlsx Primary statistics of sequencing data and assembled genomes.

Supplementary material 2 jcm.00741-23-s0002.pdf Detailed analyses of discrepant results.

Supplementary material 3 jcm.00741-23-s0003.xlsx Excel spreadsheets 3A to 3D.

Supplementary material 4 jcm.00741-23-s0004.pdf ONT and Illumina cps sequences.

ASM does not own the copyrights to Supplemental Material that may be linked to, or accessed through, an article. The authors have granted ASM a non-exclusive, world-wide license to publish the Supplemental Material files. Please contact the corresponding author directly for reuse.
