# Supplementary material for: Comparison of next generation technologies and bioinformatics pipelines for capsular typing of Streptococcus pneumoniae
Source: J Clin Microbiol. 2023 Nov 21;61(12):e00741-23. doi: 10.1128/jcm.00741-23 (PMC10729682; doi:10.1128/jcm.00741-23)

**Supplementary material 2. Detailed analyses of discrepant results.** Summary of the main steps performed for manual inspection of *cps* locus and final serotype prediction in case of discrepant results between serological typing and WGS-based approaches.

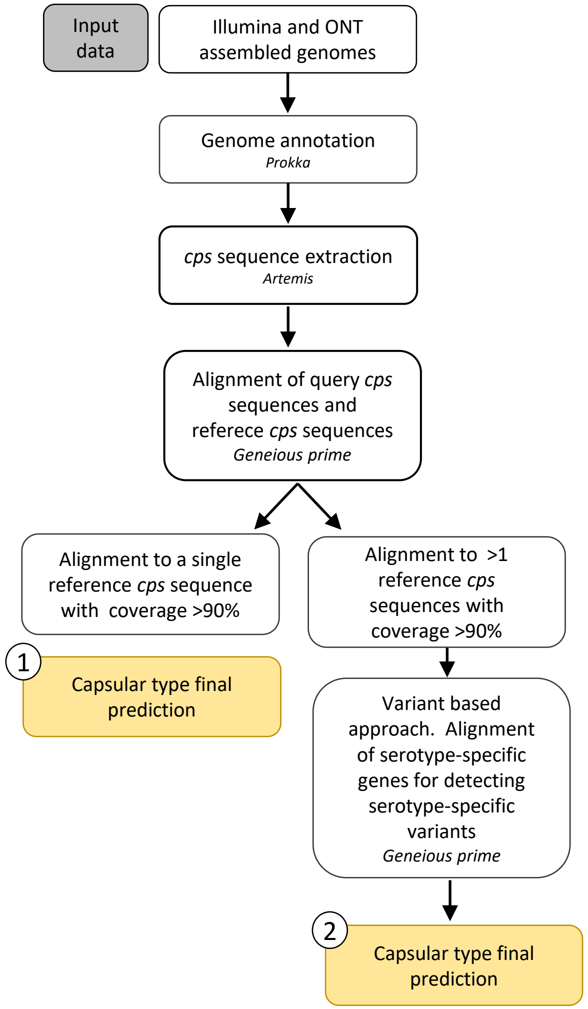

Supplement: Supplementary material 2 — Detailed analyses of discrepant results. [file jcm.00741-23-s0002.pdf]
