# Supplementary material for: Comparison of next generation technologies and bioinformatics pipelines for capsular typing of Streptococcus pneumoniae
Source: J Clin Microbiol. 2023 Nov 21;61(12):e00741-23. doi: 10.1128/jcm.00741-23 (PMC10729682; doi:10.1128/jcm.00741-23)
Supplement: Supplementary material 4 — ONT and Illumina cps sequences. [file jcm.00741-23-s0004.pdf]

Supplementary material 4A. Alignment of the *wciP* gene from reference *cps* sequences of 6A (CR931638) and 6B (CR931639) serotypes and *wciP* gene extracted from the assembled genome of the sample 2816 with Illumina and ONT sequencing data. Samples are equally coloured to the reference serotype assigned according to their similarity in the alignment.

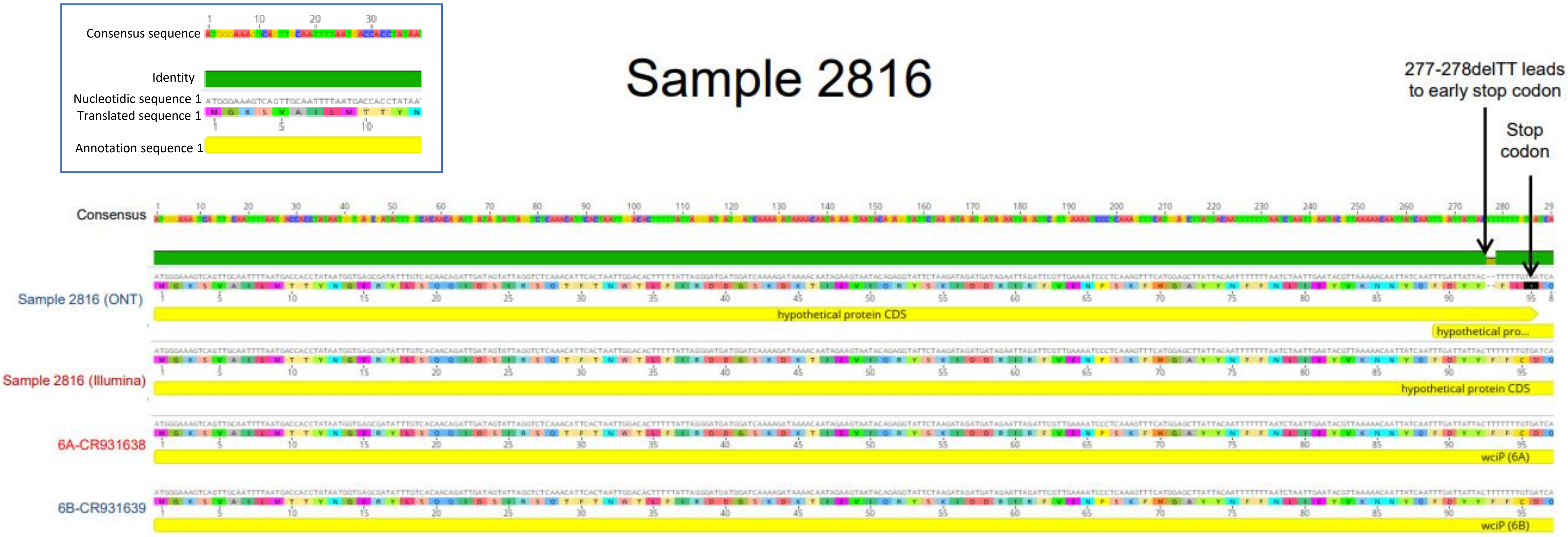

6A- Intact *wciP*
6B - 584 G>A in *wciP* leads to aminoacid substitution (Ser195Asn) which results to different rhamnose-ribitol linkages in 6B (1→4)

Supplementary material 4B. Alignment of the *wciP* gene from reference *cps* sequences of 6C (EF538714) and 6D (GQ848645) serotypes and *wciP* gene extracted from the assembled genome of the sample 3012 with Illumina and ONT sequencing data. Samples are equally coloured to the reference serotype assigned according to the variants found in the alignment.

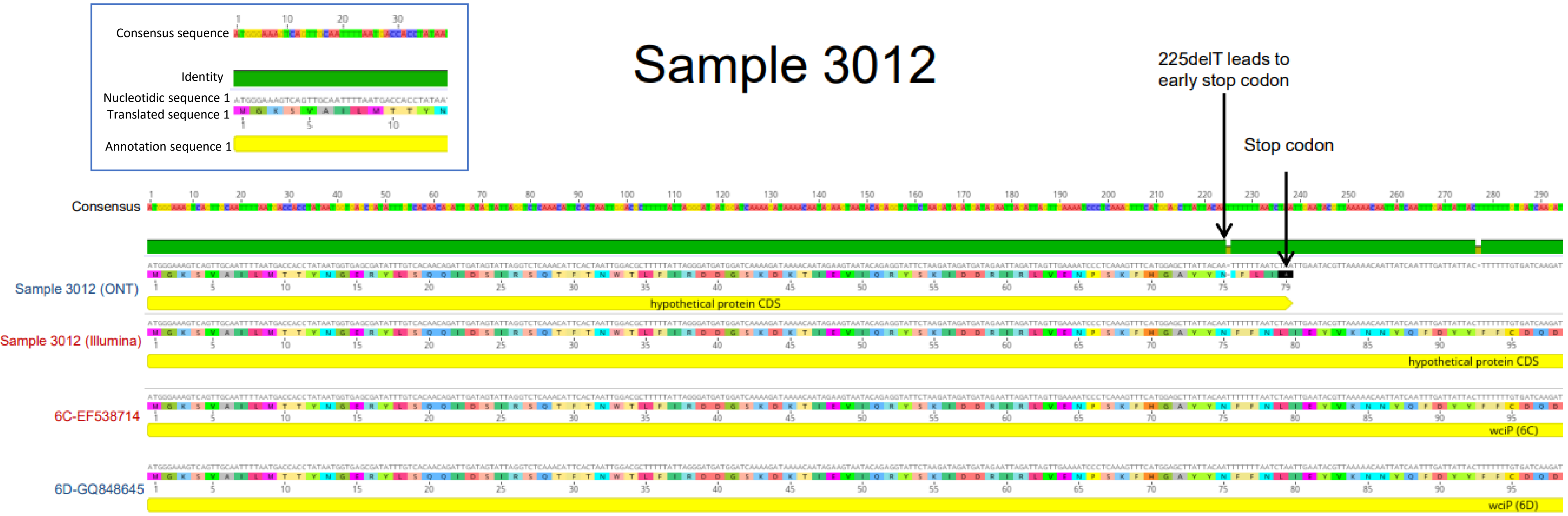

6C- 584 G>A in *wciP* leads to an aminoacid substitution (Asn195Ser) which results to different rhamnose-ribitol linkages in 6C (1→3)

6D - Intact wciP

Supplementary material 4C. Alignment of the *wcjE* gene from reference *cps* sequences of 9A (CR931645) and 9V (CR931648) serotypes and *wcjE* gene extracted from the assembled genome of the sample 3052 with Illumina and ONT sequencing data. Samples are equally coloured to the reference serotype assigned according to their similarity in the alignment.

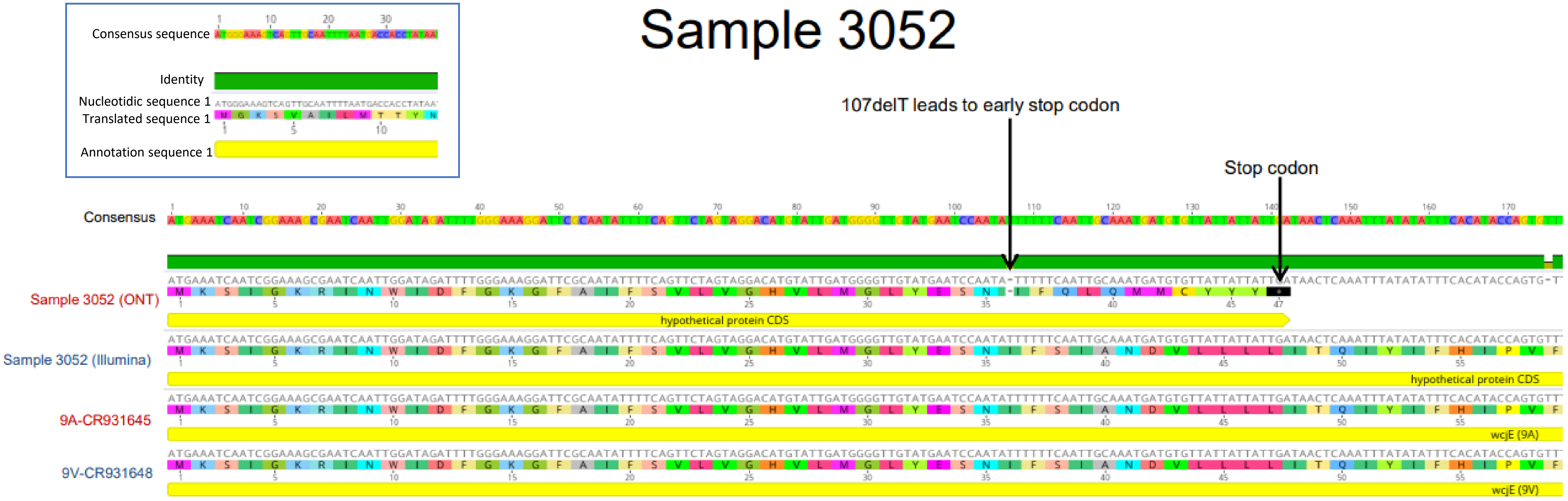

9A- 722delG leads to loss of function of glycosiltransferase which results in differences on acetylation
9V - Intact wcjE

Supplementary material 4D. Alignment of the *wcjE* gene from reference *cps* sequences of 9A (CR931645) and 9V (CR931648) serotypes and *wcjE* gene extracted from the assembled genomes of the sample 3077 with Illumina and ONT sequencing data. Samples are equally coloured to the reference serotype assigned according to the variants found in the alignment.

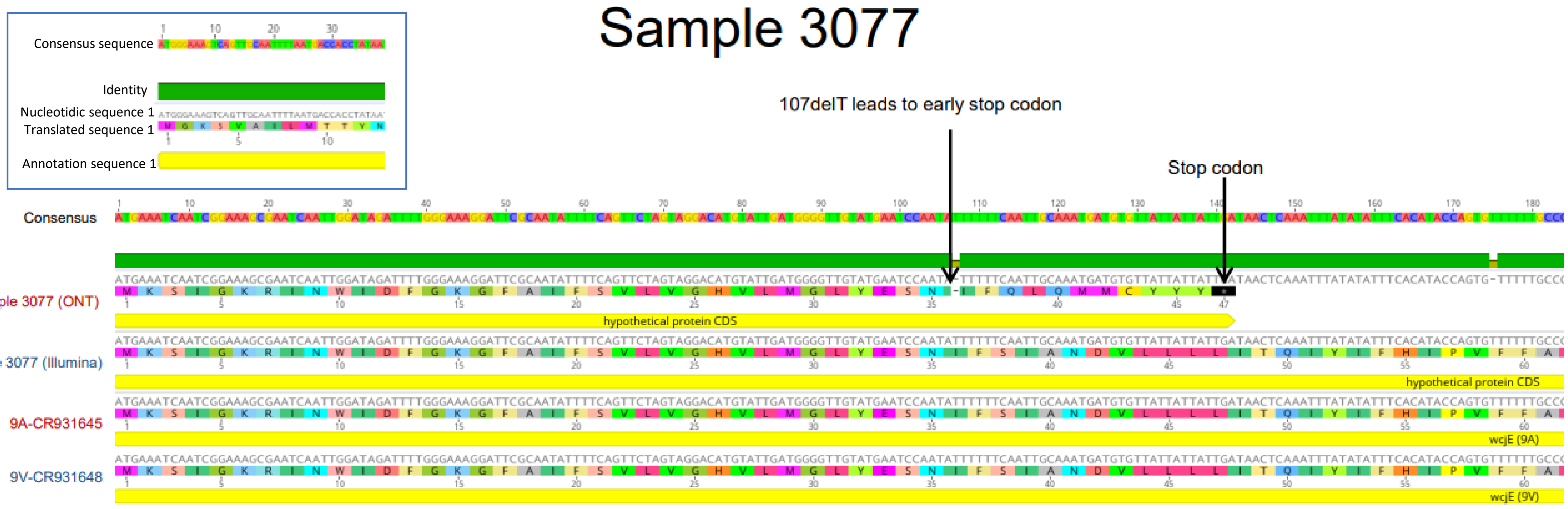

9A- 722delG leads to loss of function of glycosiltransferase which results in differences on acetylation
9V - Intact wcjE

Supplementary material 4E. Alignment of the *wcrL* gene from reference *cps* sequences of 11A (CR931653) and 11D (CR931656) serotypes and *wcrL* gene extracted from the assembled genome of the sample 2508 with Illumina and ONT sequencing data. Samples are equally coloured to the reference serotype assigned according to the variants found in the alignment.

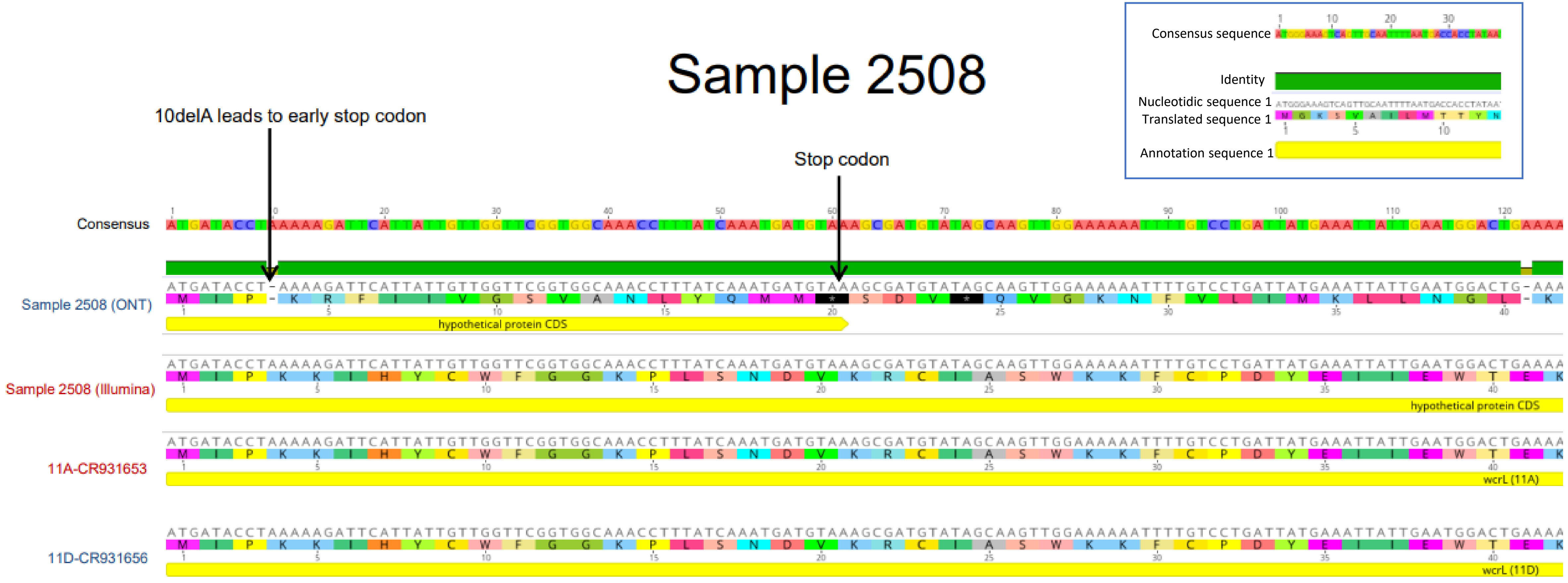

11A- Intact *wcrL*

11D - 335A>G in *wcrL* leads to an aminoacid substitution (Asp112Ser) which results in a bispecific glycosyltransferase

Supplementary material 4F. Alignment of the *wcrL* gene from reference *cps* sequences of 11A (CR931653) and 11D (CR931656) serotypes and *wcrL* gene extracted from the assembled genome of the sample 2112 with Illumina and ONT sequencing data. Samples are equally coloured to the reference serotype assigned according to the variants found in the alignment.

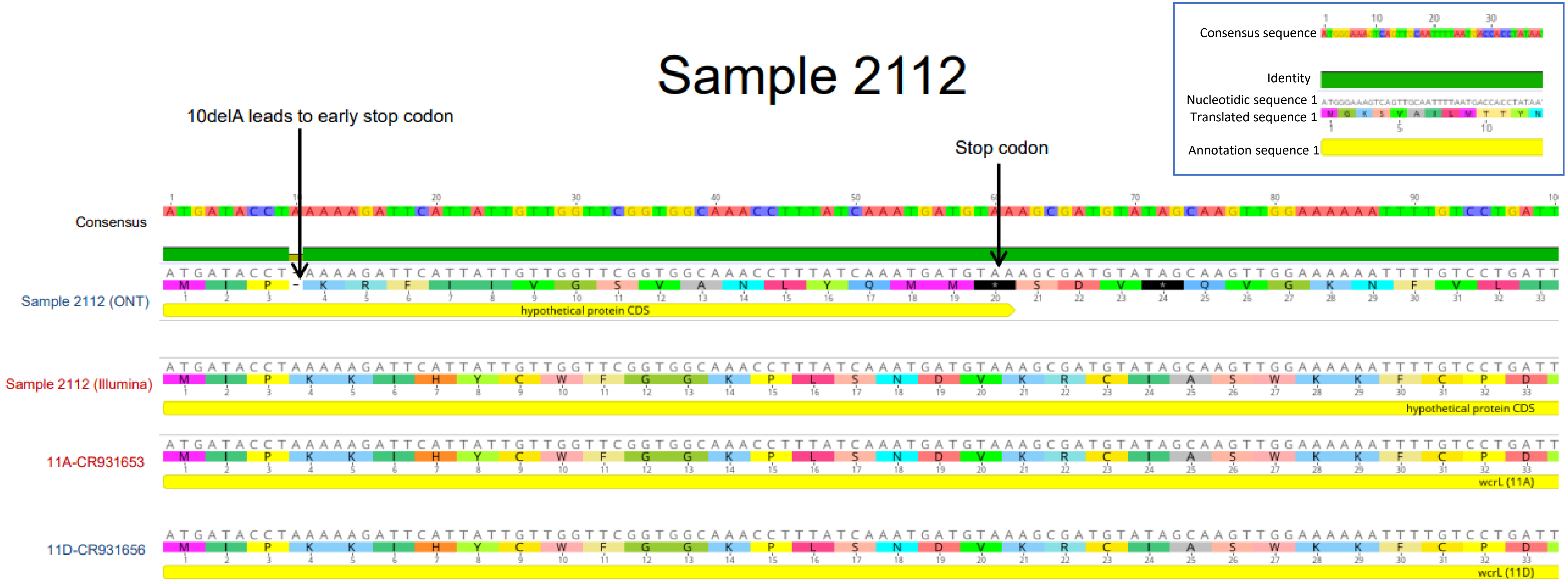

11A- Intact *wcrL*

11D - 335A>G in *wcrL* leads to an aminoacid substitution (Asp112Ser) which results in a bispecific glycosyltransferase

Supplementary material 4G. Alignment of the *wciX* gene from reference *cps* sequences of 18B (CR931672) and 18C (CR931673) serotypes and *wciX* gene extracted from the assembled genome of the sample 1963 with Illumina and ONT sequencing data. Samples are equally coloured to the reference serotype assigned according to the variants found in the alignment.

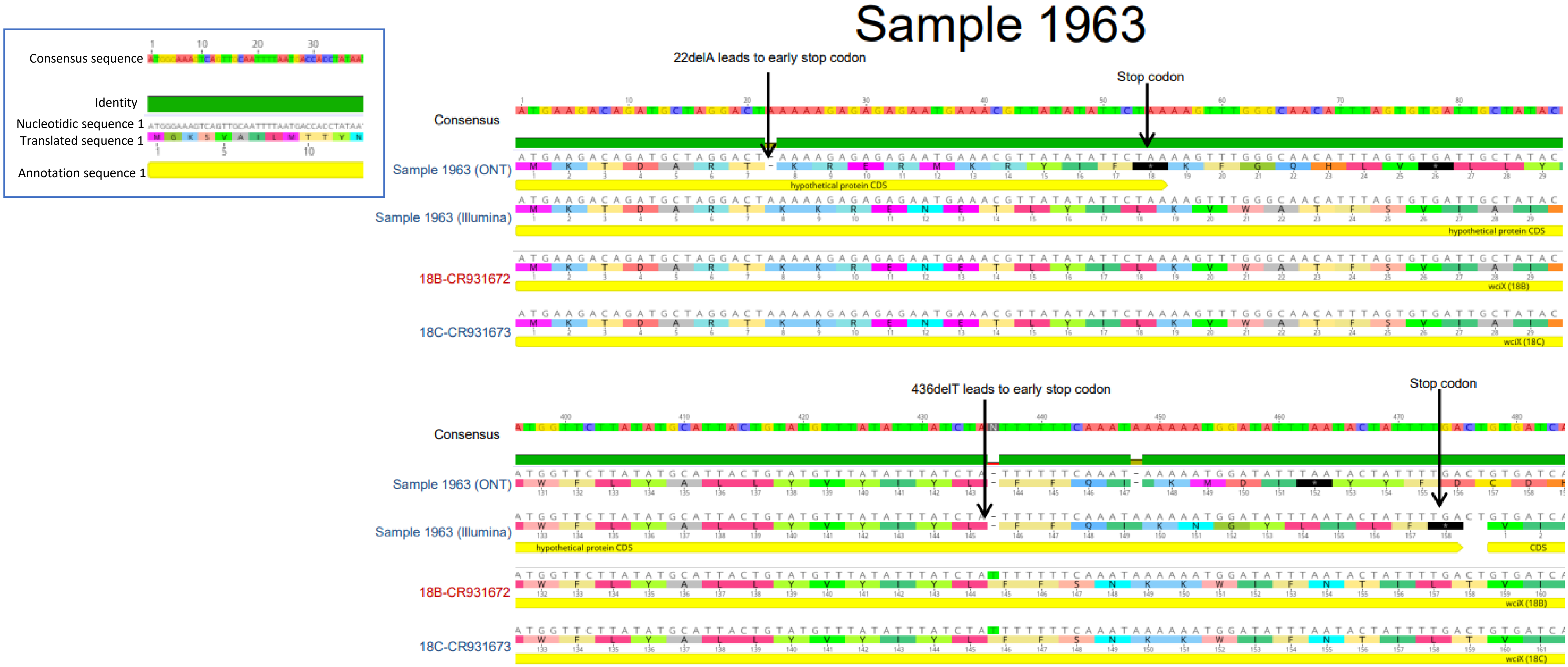

18B- 169 G>T in *wciX* leads to early stop codon which results in differences on acetylation
18C-Intact *wciX* gene

Supplementary material 4H. Alignment of the *wciX* gene from reference *cps* sequences of 18B (CR931672) and 18C (CR931673) serotypes and *wciX* gene extracted from the assembled genome of the sample 3742 with Illumina and ONT sequencing data. Samples are equally coloured to the reference serotype assigned according to the variants found in the alignment.

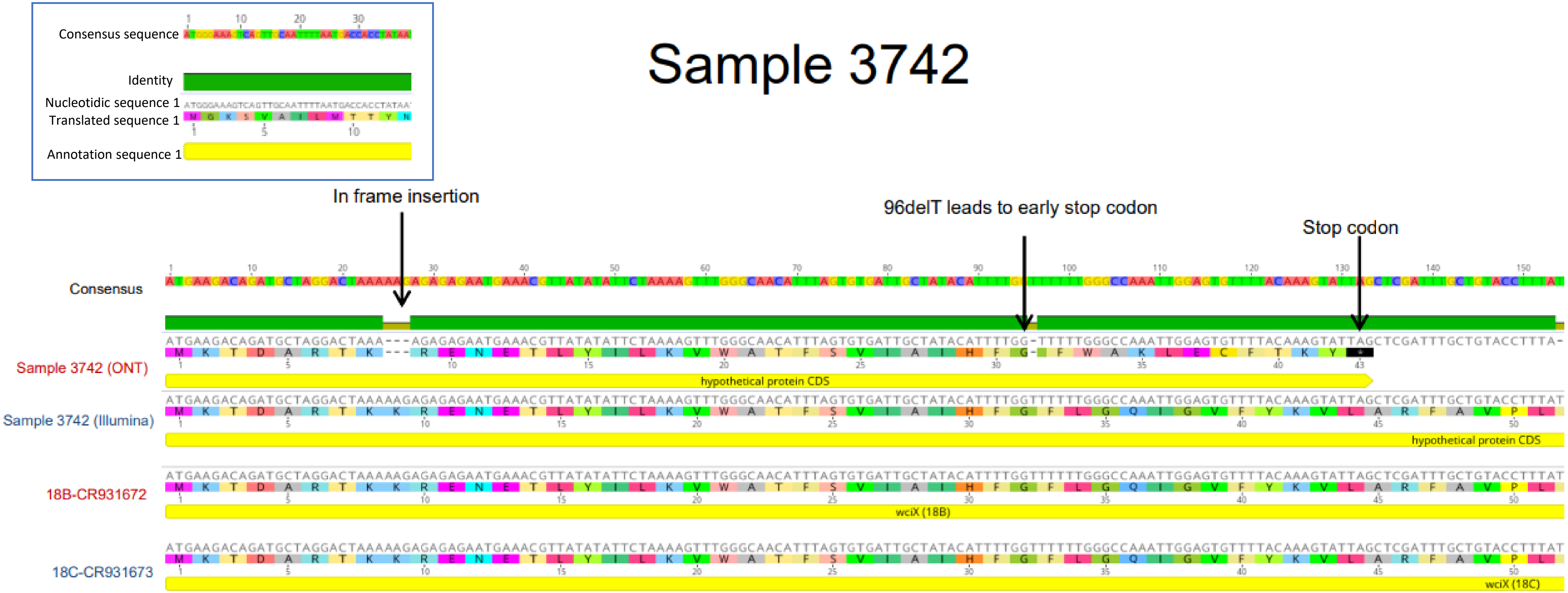

18B- 169 G>T in *wciX* leads to early stop codon which results in differences on acetylation
18C-Intact *wciX* gene

Supplementary material 4I. Alignment of the *wciX* gene from reference *cps* sequences of 18B (CR931672) and 18C (CR931673) serotypes and *wciX* gene extracted from the assembled genome of the sample 2291 with Illumina and ONT sequencing data. Samples are equally coloured to the reference serotype assigned according to the variants found in the alignment.

Sample 2291

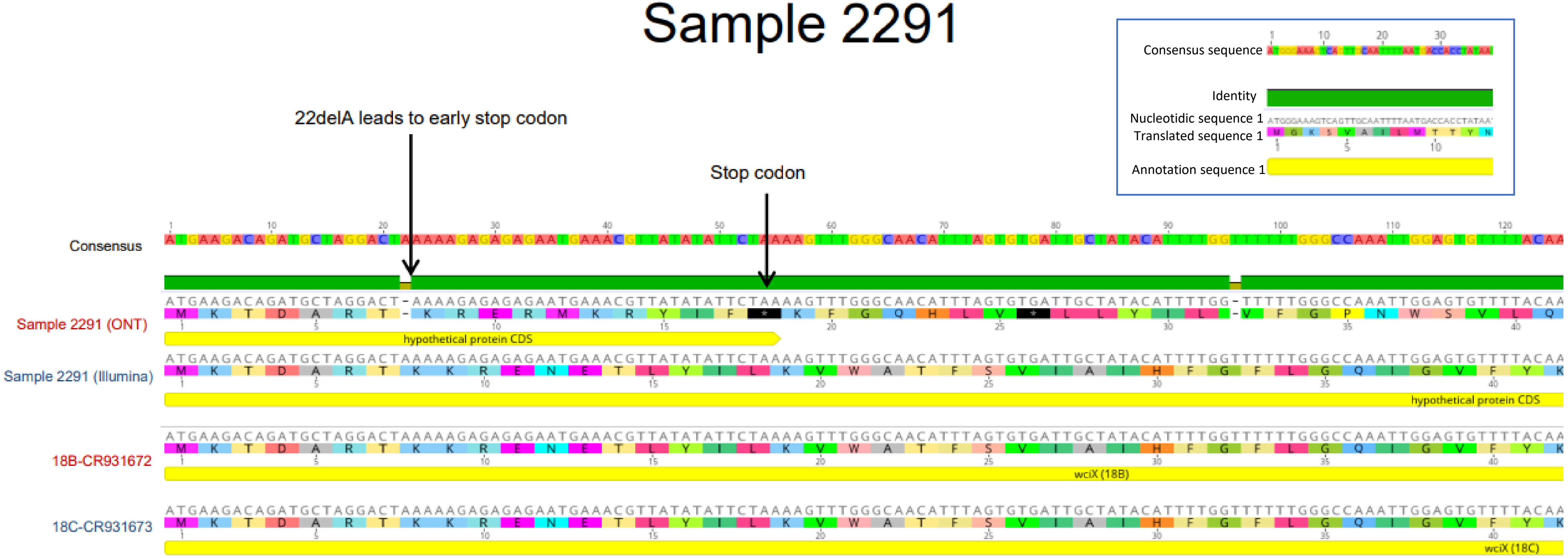

18B- 169 G>T in *wciX* leads to early stop codon which results in differences on acetylation
18C-Intact *wciX* gene

Supplementary material 4J. Alignment of the complete *cps* sequence from reference 29 (CR931694), 35B (CR931705) and 35D (KY084476) serotypes and the *cps* sequence extracted from the assembled genome of the sample 3855 with Illumina and ONT sequencing data. Samples are equally coloured to the reference serotype assigned according to their highest identities.

Sample 3855

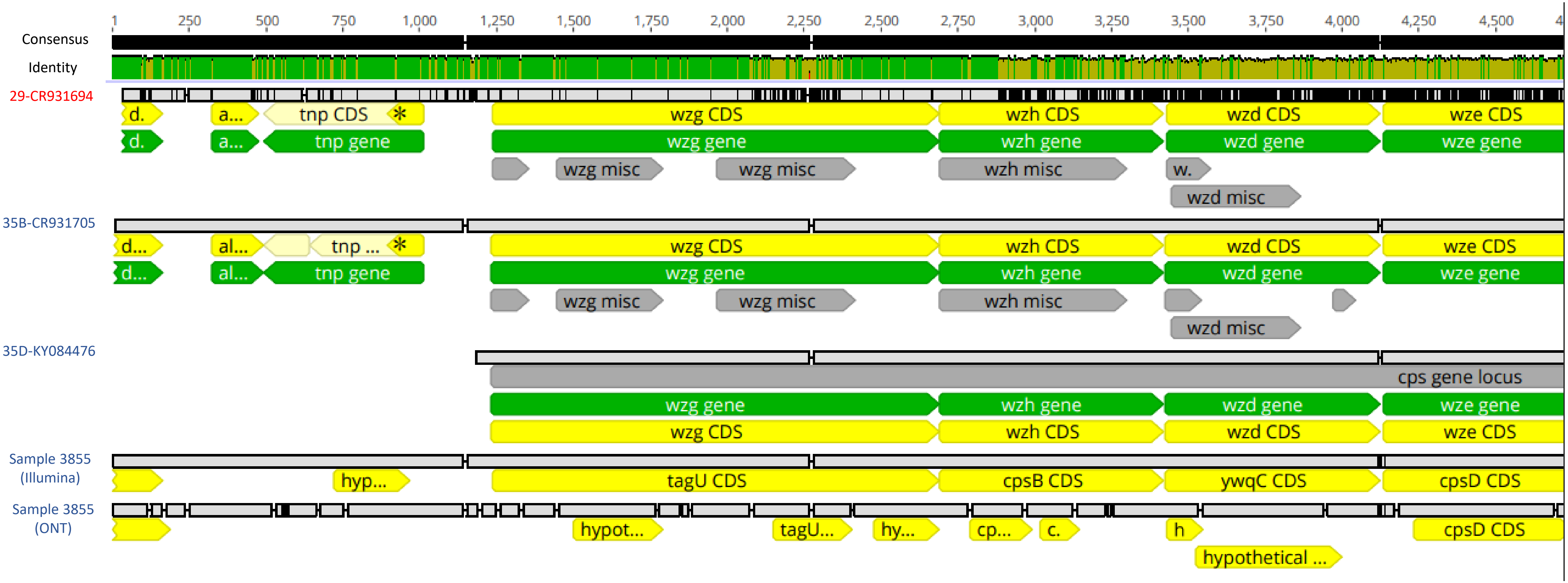

Black lines, disagreements with the consensus sequence. Higher identity of Sample 3855 to 35B/D *cps* reference sequences

Sample 3855

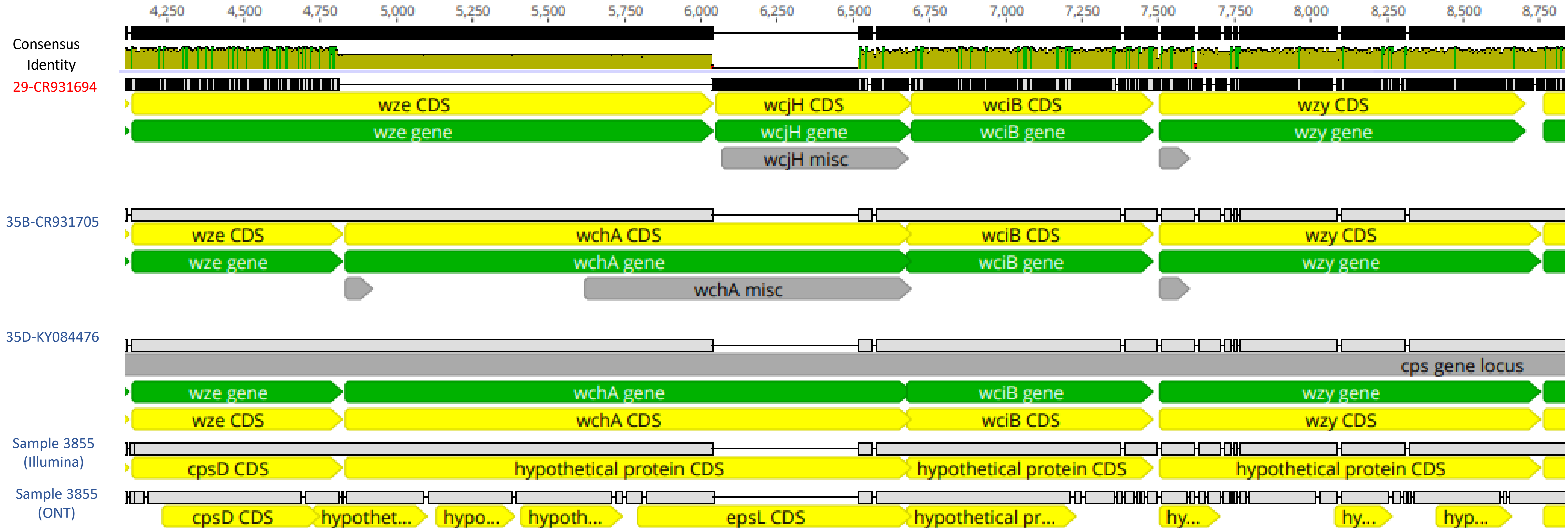

Black lines, disagreements with the consensus sequence. Higher identity of Sample 3855 to 35B/D *cps* reference sequences

Sample 3855

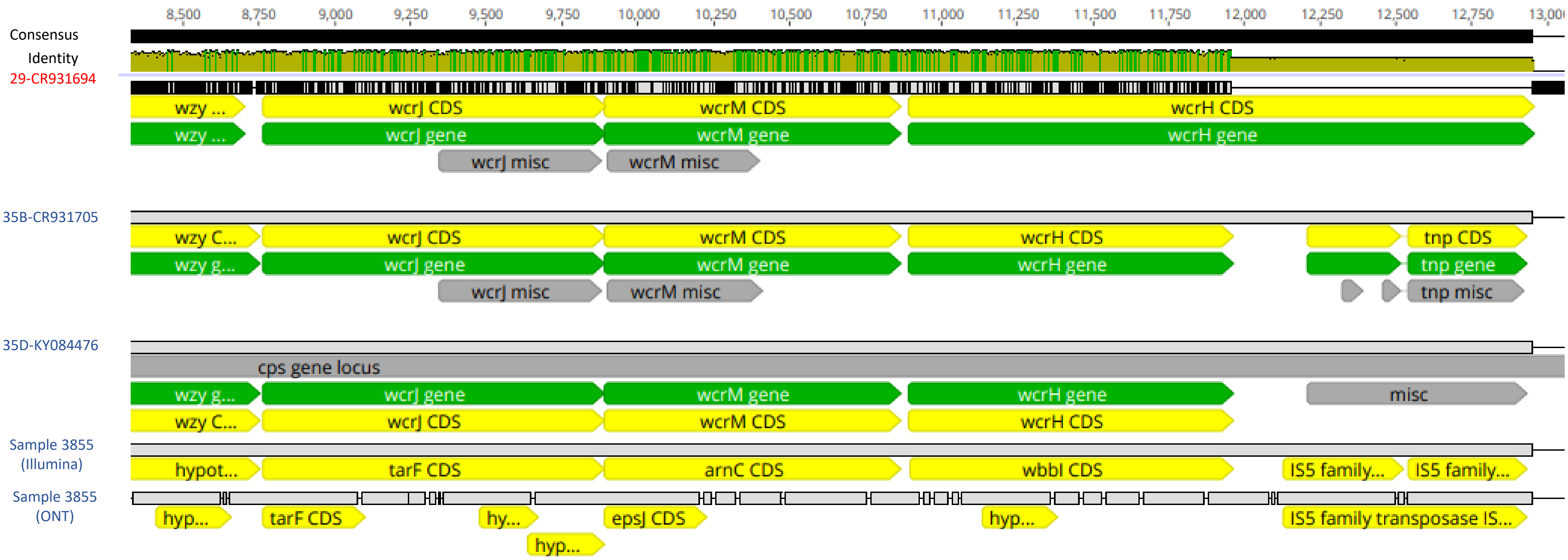

Black lines, disagreements with the consensus sequence. Higher identity of Sample 3855 to 35B/D *cps* reference sequences

Sample 3855

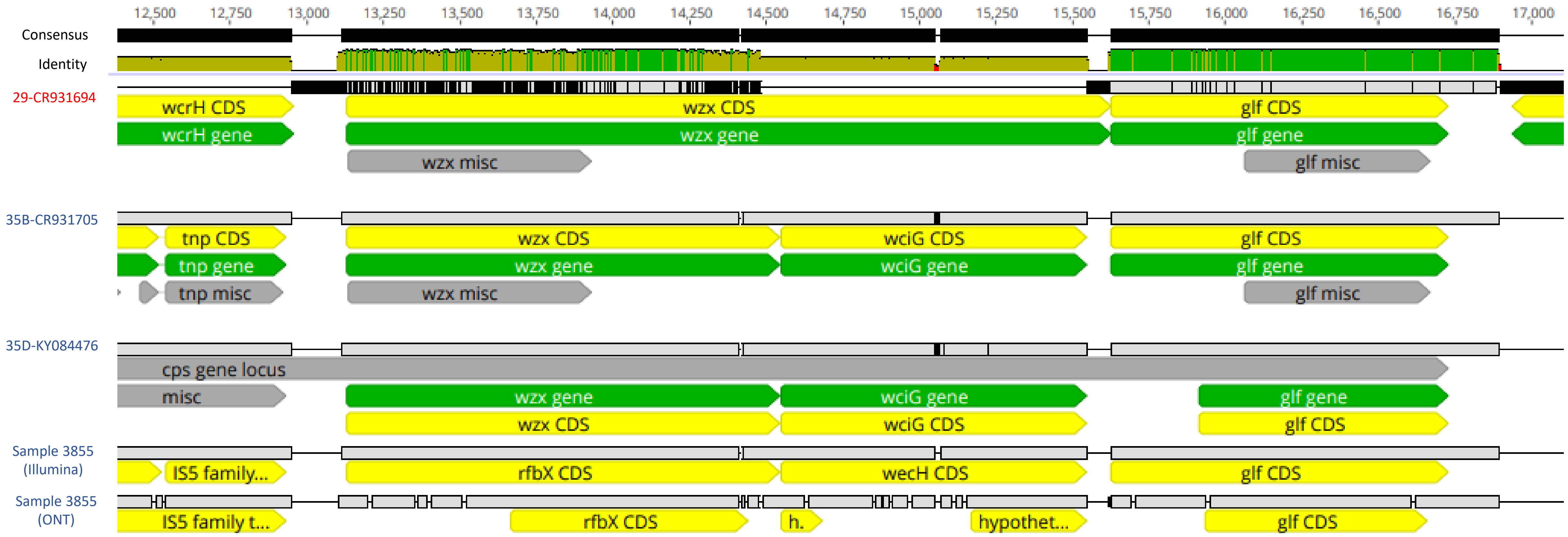

Black lines, disagreements with the consensus sequence. Higher identity of Sample 3855 to 35B/D *cps* reference sequences

Sample 3855

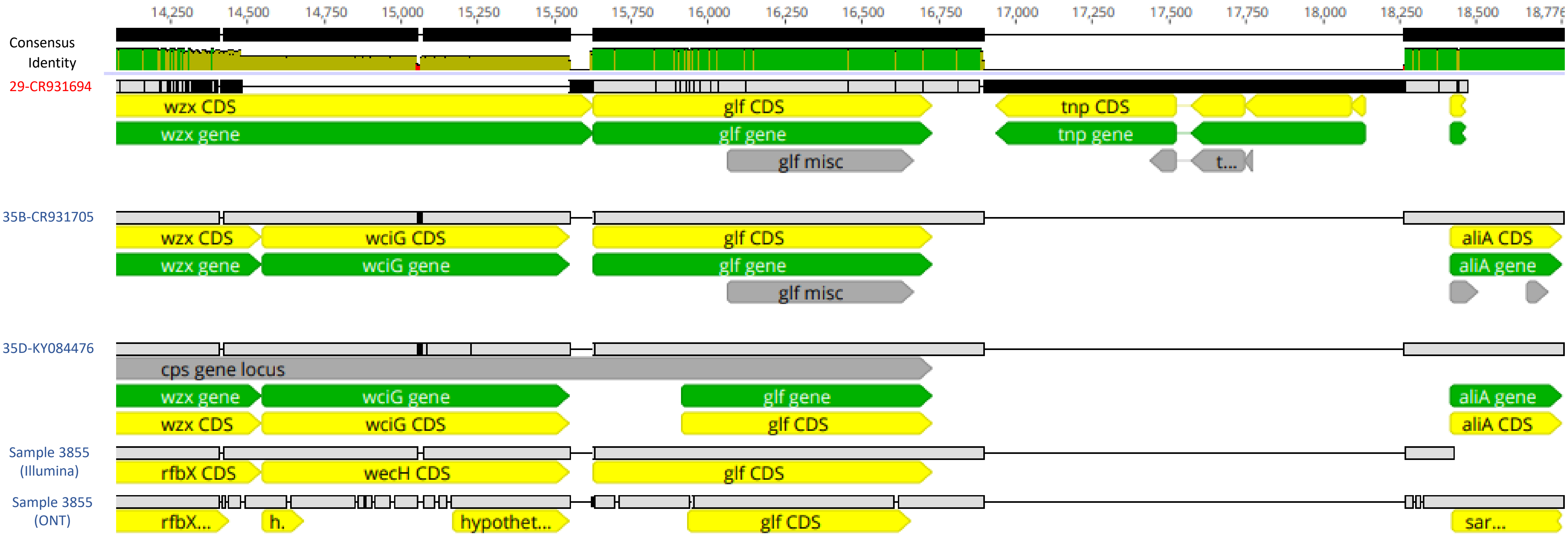

Black lines, disagreements with the consensus sequence. Higher identity of Sample 3855 to 35B/D *cps* reference sequences
